# Lectures on the More Important Diseases of the Thoracic and Abdominal Viscera, Delivered in the University of Pennsylvania

**Published:** 1845-01

**Authors:** 


### ART. II.

*Lectures on the more important Diseases of the Thoracic and Abdominal Viscera, delivered in the University of Pennsylvania.* By N. CHAPMAN, M.D., Professor of the Theory and Practice of Medicine, &c. &c.—*Philadelphia,* 1844. 8vo, pp. 384.

MEDICAL research proceeds with untiring activity. Modern times may justly boast of the successful zeal with which the accessory sciences are cultivated ; but it must be acknowledged that the practice of medicine fails as an art to make commensurate progress. This fact may be partly attributable to the greater facility with which distinction is attainable by the cultivation of some one more distinct field of inquiry, and to the greater arduousness of those exertions which require for their efficiency a judicious adaptation of means derived from many sources. Whatever be the explanation, the circumstance is much to be regretted. Great as is the value of collateral knowledge, the primary object of the physician is to subdue disease; and his duty is neglected on insufficient grounds if he spends his time inquiring " what news ?" instead of combining his resources against the common enemy. Under such convictions we examine with especial interest any respectable contribution to practical medicine ; and recognizing the world as the field of medical labour, gladly embrace any opportunity of recording the proceedings of our brethren in other countries.

The work which we now introduce to our readers represents the opinions of one of the oldest, and therefore it is to be presumed one of the most experienced physicians of America, on several important diseases especially prevalent in that country. A considerable proportion of the professors of our art may be divided into those who teach without practising, and those who practise without teaching. Among those who combine these offices are to be found some of the most valuable members of the medical community ; and the author of the work before us, notwithstanding certain striking defects in its execution, is entitled to the favorable consideration of his professional brethren. The topics introduced are too numerous to allow of more than a cursory notice, but we shall endeavour to report any peculiar opinion which Dr. Chapman may entertain, as well as any particular facts which his position in the New World may have given him especial opportunities of observing.

I. Thoracic Diseases. The affections treated under this head, are phthisis, cynanche laryngea, asthma, and angina pectoris. With the exception of the second disease, fully noticed in our last Number, we shall give some account of these in order.

*Phthisis.* In the chapter on phthisis with which the volume commences, Dr. Chapman claims for Sylvius de la Boe (A.D. 1679) the credit of first accurately describing the disease, and advances the opinion that little of importance has been added to the remarks subsequently published on the subject by Starke. He discusses the nature of the granular tubercles described by Bayle; on the one hand, differing from Laennec and Louis, who consider them essentially the same as genuine tubercles; and on the other hand, from Andral who regards them as hepatized granulations unusually indurated. Dr. Chapman considers them as tuberculoid rather than tubercular, and the result of inflammation in subjects partaking of the tubercular diathesis.

The author is a strong advocate of the independence of phthisis and scrofula:

"Contrasted with the scrofulous, it is in the first place to be stated, that with little of the external physiognomy appertaining to that state, it (consumption) has its own peculiar physical structure and aspect, among the features of which are the long delicate neck; narrow flat, chest; prominent shoulders, high cheek-bones, long arms, large hands and feet, dark hair and eyes, with lengthened and tapering lashes, thick skin and dingy complexion.

"They also differ in some particulars, in the moral and intellectual constitution with which they are associated. As soon as the strumous habit becomes oppressed by an affection of the organs of the cavities, all vivacity is exchanged for gloom, petulance and querulousness. The tubercular on the contrary retains its original qualities throughout, in all stages, and in every fluctuation.

"Further and material differences are recognizable in the two affections, the strumous being most prevalent in childhood or youth, and the tubercular in maturer age. The former too, assails both the internal and external structures, while the latter takes a centripetal direction only.

"Nor is their mode of approach the same or even bearing a close similitude. It is undeniable that scrofula in early life especially, nearly always, at first betrays itself by disorder of the chylopoietic viscera; irregular appetite; bad digestion; vitiated alvine discharges; tumid abdomen, &c. All which may be relieved by a translation to the surface. Tubercular formations have no such signs, or in their progress even manifest any disposition to metastasis, or change of position." (pp. 24-5.)

We are far from subscribing to the accuracy of many of these so-called distinctions; and when we find our author adding, as the crowning discrepancy of the two affections, a positive pathological difference, which does not exist, our distrust of the validity of his arguments becomes greatly enhanced.

"But," continues Dr. Chapman, "the great and irreconcilable distinction between these affections consists in tubercles being altogether an extraneous production, and strumous tumours, degenerations of lymphatic glands. The first is a new creation of an irregular nutrition, while the other is the diseased growth of a natural pre-existing organ." (p. 25.)

Our readers do not require to be told that all the best pathologists have long taught that precisely the same "extraneous production" exists in "strumous tumours" as in the lungs in phthisis.

Dr. Chapman has observed some striking instances of the suspension of phthisis by gestation, but he always found the disease return with aggravation after delivery, and hurry on to a fatal termination. He is of opinion that phthisis is often suspended by other diseases, such as mania, chronic eruptions, gout and rheumatism.

"There is indeed a mixed variety of the latter affections, attacking more especially the smaller joints, inducing permanent enlargements, and deposits of calcareous matter, which so strongly exercises such an influence, that it may almost be deemed a cure. Three instances of its conspicuous efficacy in this way, have come under my notice. The same is partly true of fistula in ano. Consumption and it are often coincident, the former being generally checked or mitigated by the latter. This however healing, which is very difficult to accomplish, under such circumstances, the pulmonary affection I have uniformly found to be immediately exasperated to a fatal issue." (p. 44.)

The above observation on the influence of fistula corresponds with our own experience.

On the subject of *treatment* in phthisis we find little to extract of a satisfactory character. Dr. Chapman believes life to be occasionally prolonged by the conversion of the cavity into a fistulous state, but "the alleged curability of matured tubercles is refuted by experience." The only remedy of which the author speaks with much approbation, is nitrate of potash, which he thinks very useful in allaying inordinate action, as much as half an ounce being given daily in a quart of water.

His opinions of the expedient of medicated inhalation, more especially of the vapours of iodine and chlorine, are embodied in the following passage:

"Each of these fumes I have repeatedly tried, and though I cannot say that I ever accomplished even an approximation to a cure by them, I am persuaded, that they are deserving of some attention. Moreover, the vapour of the chloride of lime has been fully used by me, which, as equally, or perhaps more efficacious, and being prepared with greater convenience, is, on the whole, to be preferred. Though this practice may not do more, it often allays cough, facilitates expectoration, diminishes or corrects purulent and other secretions, abating at the same time most sensibly hectic irritation, and hence appears the best suited to the affections of the mucous surfaces of the windpipe, and its ramifications. Yet there are cases with an excess of irritability of this structure, in which it is not tolerated, and if persisted in, is evidently productive of harm." (p. 71.)

In the treatment of hectic fever, Dr. Chapman recommends drachm doses of distilled vinegar to be given in sugared water every two or three hours. The night-sweats, which he attributes to debility of the exhalents, he thinks may be moderated by sleeping in flannel, rubbing the skin with a solution of one drachm of alum in half a pint of brandy, and administering mild diuretics such as water-melons, parsley-tea, or soda-water.

We believe the truth respecting the influence of marshy situations is pretty fairly conveyed in the remark—"It is possible that consumption might be suspended *temporarily* by the prepotency of intermittents." Milk and farinaceous diet are recommended, but asses' milk is not considered entitled to a preference, unless in the language of the celebrated Harvey, "the patient be an ass." Dr. Chapman considers the opinion of Stoll, that whenever a consumptive patient mounts a horse, he commences a journey to the river Styx, "as correct when applied to cases in which the inflammatory diathesis prevails;" but in other instances he concurs in the recommendation of Sydenham and Fuller, the latter of whom quaintly remarks that a consumptive patient should be like an Arab always on horseback.

We observe with pleasure the buoyancy of spirit with which our veteran brother closes his engagement with the gigantic foe.

"He who shall determine correctly the pathology, and indicate with any certainty the cure of this disease, will wipe away a reproach to our art, and earn a reward as much more glorious than the oaken wreath awarded by the Roman senate to a soldier on saving an individual in battle, as the preservation of the lives of thousands, is to that of one, or the triumphs of science, compared with the solitary achievements of military intrepidity or prowess." (p. 95.)

*Asthma.* The chapter on asthma exhibits the desultory character of the author's mind, but contains many interesting observations. The disease is described by him as a bronchial spasm occasioned by irritation generally of the pneumogastric, sometimes of the spinal and ganglionic nerves often followed by congestion of the pulmonary tissues, and eventually by multiplied lesions of structure.

"It were well (he adds,) had a closer attention been directed to the influence of spinal irritations in the production of the disease. Cases having unequivocally a location in the upper portion of the vertebral column, I have so repeatedly seen, that I am persuaded of its being a more common origin of it than heretofore suspected." (p. 135.)

Dr. Chapman gives some interesting illustrations of the well-known capriciousness of asthmatic attacks in regard to locality, in different individuals.

"The pure air of the country, especially in elevated positions, I have found with very few exceptions, more pernicious than that of cities, and even the suburbs of these, less propitious than the central and populous parts. Many instances have come under my own view, of persons affected in this way, who, very comfortable in the latter, were rendered otherwise in the former situation, among which, is that of a friend of mine, who can seldom walk to the edge of the city with impunity, and never goes into the country without an attack. It sometimes happens, too, that individuals may spend the day comfortably in any rural position, though on the approach of evening, are unavoidably seized. Three or four instances of this kind I have known, and of which, there is now living in Baltimore, a gentleman by whom I am informed the fact is strikingly illustrated in himself. Close to the town, he owns a villa remarkable for its general healthiness, at which he has not slept for many years on this account, escaping however, all intimations of an attack during the day.

"Moreover, positions nearly contiguous in the heart of a city, may vary widely in this respect, even the several stories of the same house. Each of these statements at least, is verified by one case which was the subject of my care. Called to visit a young lady from the south, having a violent paroxysm of the disease, I was told that she derived an immunity from it during a recent residence in Paris, by selecting a medium story in a hotel in a particular portion of that city, and that whenever she quitted the apartment, a paroxysm soon came on, from which she was speedily relieved on returning to it. Curious to make the experiment, I was seconded in the desire, by her own anxiety to change her lodgings, where she had severely suffered, and in a very short time she went to another house in the neighbourhood, in which she entirely escaped for several months. Compelled, however, to leave it, she fixed her abode at the distance of a hundred yards, in a street no less thickly built, and here, she had scarcely any exemption for weeks. On her moving into a different quarter of the city, I witnessed a complete verification of the statement she had made me. As long as she occupied the chamber on the second floor, she was almost nightly harassed by renewals of attacks, which were presented by her sleeping in the room above. Even by dining below, her respiration was on several occasions seriously affected.

"In an individual whom I attended, the disease was twice brought on by sleeping in a room where a rose-bush in bloom was placed; in another, by a similar exposure to some pots of hyacinths; in three girls, by the emanations in the process of manufacturing cigars; and there was formerly a student in the University of Pennsylvania, who assured me that he could not weigh out a dose of ipecacuanha without being affected." (pp. 133-4.)

In the treatment of asthma, the professor has found the juice of the poke (phytolacca decandria,) serviceable, but he evinces a superstitious dread of lobelia, a remedy which in this country we have continually seen safely and usefully employed.

The following brief statement might form the text for a most valuable practical dissertation on the successful treatment of asthma. We are assured by long and extensive experience that the rational cure of many cases of asthma as well as of numerous other chronic diseases, is only to be found in the complete alteration of the patient's mode of life, and especially in the adoption of new habits of temperance, of regular and great bodily activity, and in the prosecution of that form of hygienic discipline commonly known by the name of "hardening." It is on this principle, in a considerable degree at least, that *hydropathy* proves beneficial in a certain proportion of cases.

"During the late war when the volunteers of Philadelphia were called out, and encamped several months, part of the time in winter, two individuals of my acquaintance, who previously had been dreadfully harassed by the disease, entirely escaped." (p. 148.)

*Angina pectoris.* The article on angina pectoris possesses considerable interest; for although containing little which will be novel to those who are conversant with the 'Cyclopædia of Practical Medicine,' or with the writings of Butter, Macqueen, Elsner, &c., still it is obvious that ignorance of much that has been written on the subject places Dr. Chapman in the position of an original observer, and thus adds force to opinions undesignedly corresponding with those of other writers. He considers the disease as "a species of neuralgia, commencing for the most part in the pneumogastric nerve, and spreading in different directions, as other nerves may become involved." He considers it probable that "the immediate cause of the irritation of the nerves, inducing the neuralgic condition, giving rise to the subsequent phenomena of the disease, is, in many instances at least, derived from irregular gout." (p. 158.)

In order to do justice to the professor's theory and practice, we will quote two of the cases which he has recorded: the first we would characterize as an example of a lucky hit; the second, we fear, is only valuable as a beacon to warn against the perilous shoals of the "wine-whey and ammonia" system.

"*Case* I. A distinguished member of the bar, aged 57 years, of slender form, and temperate habits, to whom I was called in the night during the winter of 1811. The moment I entered his chamber, I recognized all that sort of distress which characterizes this disease. He told me that he had for several years previously been subject to attacks of angina pectoris, for which he had been treated by Dr. Kuhn and Dr. Wistar. His pulse being active, with great agony in the region of the heart, he was bled copiously, and suspecting gout, though assured he was not liable to it, sinapisms were applied to the feet, and the carbonate of am-

42296

monia, with wine-whey, freely administered. In the course of a few hours, arthritic swelling seized on the knee-joint, with the occurrence of which all other uneasiness instantly subsided. By such a course of management, as will hereafter be detailed, he recovered his health, never having afterwards an attack either of angina pectoris or gout." (p. 159.)

"*Case* IV. In the spring of 1824, I was requested by Dr. Physick, to visit with him, a gentleman from the country, aged about 50, who informed us that for several years, he had been sorely afflicted by a complaint, considered by all the physicians whom he had consulted, as angina pectoris. The history given us of his case, embraced most of the prominent features of that affection. He told us that attacks of it were of very frequent occurrence, and brought on by the slightest causes, from one of which he was then suffering partially. As the ordinary practice had utterly failed, we determined to treat the case as irregular, misplaced gout, and with a view of drawing it to the extremities, employed the customary revellents, with carbonate of ammonia, and wine-whey. On the third day of our attendance, the gout became fully fixed in the elbow-joint, and in every other respect he felt perfectly well. Most unhappily, however, having omitted the remedies during the night, he arose in the morning to be shaved, and while the barber was occupied in that office, getting very angry with him on account of his awkwardness, he insisted on performing it himself, (as he had regained the use of his arm, from the cessation of the arthritic swelling.) But scarcely was the razor in his hand, when he complained of sickness of stomach, with excruciating pain in the left side, and sank lifeless on the floor." (p. 161.)

II. Abdominal Diseases. In the section devoted to abdominal diseases, the author exhibits a just appreciation of the great importance of the pathological relations of the digestive organs. To whatever disease, acute or chronic, he is called, he rarely omits to ask himself the question, what concern has the stomach with the case?

*Gastritis.* The description of gastritis evinces much accurate observation, and the treatment recommended is in most respects judicious, but we regret the absence of any sufficiently distinct description of a class of cases by no means unfrequent, in which gastritis is unaccompanied by its appropriate symptoms, in consequence of contemporaneous disturbance of the brain; which although only functional is too easily mistaken for cerebral inflammation. One of the most valuable means of diagnosis is the aggravation of the symptoms induced by the use of purgative medicines; but such aggravation, if overlooked, may prove speedily fatal. Some eminent French authorities attach little importance to the indications afforded by the tongue, Andral considering that there is no constant relation between its appearances and the states of the stomach, and Louis still more decidedly expressing a similar opinion. Our author considers such statements "precipitate and indiscriminate, partaking largely of the character of ultraism, which belongs to the school of Paris;" and in this sentiment we agree. We suspect that the failure of the tongue to be affected by important derangements of the stomach arises mainly from the interruption of ordinary sympathies in consequence of some overpowering impression on the nervous system. On this theory we can explain the fact, that yellow fever with an unaltered tongue is generally fatal; and can understand why we should regard a moist tongue with intense thirst, or a peculiarly dry tongue without thirst, as of evil omen.

In cases of *ulceration* of the stomach, Dr. Chapman has known a fatal result preceded by vomiting "of black blood or dark flocculose or granulated matter, to the amount even of eight gallons in three days."

*Cancer.* We can, from repeated observations, bear testimony to the truth of the assertion that cancer of the stomach may proceed "to a great extent without being betrayed by any very serious local suffering or constitutional depravation."

The quasi-epidemic prevalence of chronic, and even organic diseases has often been noticed, and most physicians of experience have had their attention called to the question by events in their own practice. The true bearings of such events are for the most part unknown: the occurrences are often, unquestionably, accidental. Dr. Chapman's attention has been called to the prevalence of cancerous affections in a similar point of view.

"Not a little curious is it that the affection appears to be of wider prevalence at particular seasons, even to the observance in a degree of an epidemic character. During the month of April, 1832, I saw eight cases of it absolutely demonstrated by autopsic inspections, and such occasional occurrences, though not to an equal extent, have at other times been noticed by me, without any obvious reason for the phenomenon." (pp. 200-1.)

While commenting on the commonly admitted fact, that griefs and anxieties contribute to the development of cancer, Dr. Chapman adduces the case of Napoleon in evidence. He says that the harassments and exasperations to which he was exposed at St. Helena developed and envenomed the disease to which he fell a victim: "the records of time," he adds, "we shall in vain search for any similar instance of wanton and barbarous cruelty." Dr. Chapman might have ascertained that the symptoms of Napoleon's disease commenced as early as the Russian campaign in 1812; and had he known this, his readers would have had little to regret in the loss of some splendid specimens of maudlin sentimentalism and mock sublime, which disfigure this portion of the work. The occurrence of occasional inconsistencies of this kind in American writers betokens the still immatured literary taste of our transatlantic brethren.

*Dyspepsia.* After a description of the principal causes of dyspepsia, such as "deleterious drinks, gross animal food, and all the things included in the term dessert," we meet with the following sentence, which is too characteristic to be omitted. Our American friends are said to be very sensitive at being "shown up," at least by strangers: we hope, for the sake of our good doctor, that he lives beyond the region of tar and feathers. Our reason, however, for quoting the passage is the important truth it contains,—a truth, we admit, with shame and confusion of face, to be not altogether unexemplified, in a small way at least, on the soil of the "old country." As a general rule, however, we hope "our people" do find time "to pick their bones and chew their scrapings."

"Detrimental as these articles may be, they are rendered still more so by the incongruous mixtures of them, several of the most discordant qualities being taken together, creative of all sorts of contentious movements in the stomach, a tumultuous uproar always ending in mischief, and above all by the rapidity with which they are swallowed. 'Bolting' is the term used to express the hurried manner of eating, in which it is said we excel every other people, and perhaps truly, owing in part to our eagerness to get on in whatever we undertake, and still more to the quantity our tables supply to be disposed of. As practised in Europe, our people cannot spare time to pick bones, and leisurely to chew the scrapings." (p. 212.)

Tobacco is denounced as a fertile source of dyspepsia; and we were delighted to meet in Dr. Chapman's pages with the following account of its deleterious effects. On this subject, an American physician may truly speak, *ex cathedra*. Although we cannot as yet pretend to compete with Americans in the practice of this disgusting vice, we see too much of it, not to know that, even in the small way here perpetrated, it is almost as injurious to the health of our young men as it is nauseous to the senses of their unsmoking victims. We almost wish they would, like Dr. Chapman's senator, come to "chewing and snuffing as well as smoking," that the full abomination of the thing might cure itself.

"By a member of congress from the West, in the meridian of life, and of a very stout frame, I was some time since consulted; he told me that from having been one of the most healthy and fearless of men, he had become 'sick all over and as timid as a girl.' He could not even present a petition to congress, much less say a word concerning it, though he had long been a practising lawyer, and served much in legislative bodies. By any ordinary noise he was startled or thrown into tremulousness, and afraid to be alone at night. His appetite and digestion were gone, he had painful sensations at the pit of his stomach, and unrelenting constipated bowels. During the narrative of his sufferings his aspect approached the haggard wildness of mental distemperature. On inquiry I found that his consumption of tobacco was almost incredible by chewing, snuffing, and smoking. Being satisfied that all his misery arose from this poisonous weed, its use was discontinued, and in a few weeks he entirely recovered.

"Distressing as was this case, I have seen others, from the same cause, even more deplorable. Two young men were in succession brought to me for advice, whom I found in a state of insanity, very much resembling delirium tremens. Each had chewed and smoked tobacco to excess, though perfectly temperate as regarded drink. The farther account given me was, that early in life, adopting this bad practice, it grew with their growth. Dyspepsia soon occurred, attended by great derangement of the nervous system, and ultimately the mania I have mentioned. But I have also seen the same condition very speedily induced." (pp. 214-15.)

The essential differences between inflammatory irritation and atonic dyspepsia are but indistinctly recognized, and the treatment is consequently discussed obscurely and inefficiently. Still we may fairly appropriate to the author the merit of having done better than others what few have done well. The treatment of this disease will continue to be an opprobium, until practitioners endeavour more carefully to distinguish between these varieties of the malady.

Dr. Chapman continually refers to gastric uneasiness, as though it were an essential symptom of dyspepsia; yet, every experienced practitioner must know that such is not the case. There is, at least, one distressing and not uncommon form of indigestion unattended with obvious uneasiness of stomach; the whole discomfort seeming as though dependent on the condition of the brain. The sufferer too well to be pitied, but too ill to be endured, can take scarcely any food without inducing an indescribable feeling of mental depression and inefficiency. We believe that, in such a condition medicines can accomplish little, and that the remedy is social cheerfulness and muscular exercise kept within the limit of pleasure. To such patients "vivite læti" is the great rule of health.

Dr. Chapman's experience leads him to believe that dyspepsia is occasionally induced by hemorrhoids:

"Two cases from several more which I might adduce, may adequately illustrate this point. My attention was particularly called some time since to that of a middle-aged gentleman, who had suffered several years so severely from dyspepsia that even his life was deemed in jeopardy. The disease was exceedingly well marked. Learning that during the whole time, with scarcely an intermission, he had been annoyed with painful hemorrhoids, these were removed, and almost immediately he recovered his health.

"Even a worse case in a gentleman somewhat younger, subsequently came under my care. To great emaciation and hectic irritation was added a condition of stomach which almost precluded the retention of nourishment of any description. More or less of whatever was received, soon returned by a sort of spasmodic regurgitation. Every evening he became excessively oppressed, and was only relieved by vomiting of a very large quantity of a corrosive acid fluid; which on different occasions, being deposited on the grass of his lawn, this was as completely killed as if by salt and water poured on it. No course of treatment I could devise, proved of any essential service to him, till having ascertained that he had long been affected with piles, a circumstance previously concealed from me, they were extirpated and with the happiest effect." (pp. 216-7.)

*Gastrodynia.* In the treatment of this symptom of dyspepsia, milk is a favorite remedy with Dr. Chapman.

"Milk is sometimes very effectual. Two cases, especially, have come under my notice, in which its utility was remarkably displayed, the spasms being more speedily and effectually relieved by a copious draught of milk than by opiates or any other means. My friends, the late Mr. Dallas, secretary of the treasury, and the late General Williams, of the engineer corps, whom I attended for many years, I have again and again seen cured of this agonizing affection, in a moment, as it were, by swallowing a pint of milk. Not a few other instances could I relate where the remedy proved efficacious. (p. 229.)

It would appear, however, that this favorite remedy has sometimes its inconveniences, "especially when it accumulates in large masses of a cheesy nature, productive of gastric oppression, or unrelenting obstruction of the bowels with the alarming consequences of such conditions."

"Three very remarkable instances of the kind I have met with. Many years ago I had brought to me a mass, which, in length, breadth, lobulated structure, and general aspect, so closely resembled the pancreas that it might at a glance have been mistaken for that organ. On tearing off a part of the integument, which was a coating of coagulable lymph, I found the interior to consist of compact cheesy matter. As an explanation of the phenomenon, it was stated by Dr. Tydiman, my informer, that several months before, on a journey to South Carolina, his coachman, in whom it occurred, drinking copiously of milk, was soon after seized with colic, which though relieved, so many of the symptoms of hepatitis occurred that the case was treated under such an impression. By a violent effort of vomiting, the mass was brought up, convalescence commenced, and an entire recovery ultimately took place. The preparation is in the museum of the Pennsylvania University." (p. 241.)

Our author's pages supply us with many other examples of rather odd remedies. He has experienced in his own person great relief from sick headache by the use of lemon juice. Hard cider taken in the morning fasting is much employed in America for the same purpose.

"Constantly have we reports of the most discrepant sorts of nourishment agreeing with dyspeptics, partly to be referred to individual idiosyncrasies, though much more to the pathological state of the case. The late Professor Wistar informed me that he had for a long time ineffectually endeavoured to relieve an opulent merchant of this city, who was very speedily cured by drinking copiously of *sour beer*, such as had been utterly condemned by the brewers as spoilt and unsaleable. An eminent lawyer got well of a very inveterate attack, previously attended by the late Professors Rush and Physick, by living exclusively on raw turnips, and the latter was in the habit of relating another case where raw cabbage produced the same effect.

"During nearly a whole winter I had under my care, in consultation, a most distressing attack of the disease, proving utterly intractable to the regular remedies, which the next summer promptly disappeared by the person subsisting on the *morilla* or *sour pie cherry*. Nor is this the only instance of which I have heard of cures ascribed to tart, and perhaps unripe fruit of several kinds, and one especially from Professor Hodge, to apples.

"Not many years ago, I saw with Professor Jackson, a gentleman from North Carolina, who after baffling all our efforts, recovered on a diet of fat *roasted pork;* and a second with Dr. J. Rhea Barton, in an advanced stage of the affection, with a state of stomach so irritable that nothing we directed could be retained, which immediately yielded to *wheaten mush* and *vinegar* largely and eagerly consumed." (pp. 248-9.)

What "wheaten mush" may be, we really cannot say; and here as elsewhere we wish our worthy doctor had made use of language more generally intelligble.

Some interesting examples are given of pulmonary disease arising from disorder of the alimentary canal and other parts.

"In 1820, I was called into consultation with the late Dr. Monges, to a boy, who with most of the symptoms of the preceding case, particularly the copiousness of purulent expectoration, was so much reduced that the portions of the bones on which he rested, had protruded through the integuments in several places. Discovering that his abdomen was tumid, his tongue furred, his appetite capricious and depraved, and that he was subject occasionally to convulsions, I was led to expect the existence of worms, and under such an impression, anthelmintics were administered. In less than one week, sixty-eight lumbricoides were evacuated, and from that moment he became convalescent and rapidly got well.

"Many years ago, a case occurred in this city which attracted great attention. The son of one of our distinguished citizens, swallowing a pin, was soon seized with gastric irritation, followed by cough and other pulmonary affections. These gradually increased till purulent expectoration and hectic fever took place, leaving no doubt of confirmed phthisis and a fatal result. But, at this moment of despair, a small abscess arose in the groin, which, on being opened, the pin was found in it and extracted, it having travelled down through the cellular tissue to this point. Convalescence speedily ensued, ending in a perfect recovery. The subjects of these cases are all still living and in robust health." (pp. 236-7.)

*Diarrhea.* We have some interesting notices of this malady, part of which we shall extract.

"To chronic fluxes of the bowels certain sections of our country are singularly liable, especially Richmond in Virginia, and New Orleans. Cases of it I have annually from each of these cities, and am assured that it is one of the most terrible of their maladies. No age, sex, or condition of life is entirely exempt from it, though it rarely occurs before puberty. What occasions it is not ascertained; nothing peculiar about Richmond exists to which its production can be referred; but at New Orleans the popular notion connects it with the use of the turbid waters of the Mississippi.

"Diarrhea of a somewhat different kind appears to be hardly less frequent among our Eastern population, especially that of Boston, the source of which is as little intelligible. But the individuals whom I have attended with it, in their passage through this city to the South, all concurred in stating that the attacks were ushered in as dyspepsia, followed, after a long interval, by the bowel affection, then cough and other pectoral symptoms, marasmus, hectic fever, &c., imitative of phthisis pulmonalis." (p. 279.)

The remedies employed are bleeding, general or topical, if inflammation be present, blisters; subsequently ipecacuanha emetics, warm salt water baths, Dover's powder, with torrefied rhubarb, alum (a remedy in this country perhaps unduly neglected), with sulphate of iron, acetate of lead, mercury, nitromuriatic acid, nitric acid, with opium and camphor mixture, and sometimes diluted vinegar. In the subsequent periods, copaiba, creosote, cubebs, nux vomica. When there is much debility of the exhalents, astringents, such as galls, tannin, rhatany, and the dewberry root (rubus trivialis.)

"The physician-general of the British forces in Canada told Dr. Chapman 'that his wife having suffered from diarrhea for a long period, during which she had visited Europe, and received there the best medical advice without avail, was finally cured by living entirely on oranges, to which she was prompted by an urgent desire.' Yet, generally, fruits disagree, or prove as injurious as the common vegetables. Perhaps no article of food more uniformly agrees with the patient than buttermilk." (p. 291.)

*Constipation.* We have several interesting remarks on this symptom also.

"From ten drops of the radical tincture of colchicum, repeated several times in the twenty-four hours, and persisted in for some time, as much may be anticipated with a view merely to the restoration of the lost susceptibility of the bowels, as from anything else within my experience, rarely, indeed, having seen it to fail. It is essential, however, to its success, that the dose be small, and this is a precept to be observed in relation to all medicines in this form of constipation. The object being attained rather by gradual insinuation than by forcible impression." (p. 299.)

The laxatives from which Dr. Chapman has observed advantage are, extract of the butternut (juglans cinerea), the bile of the ox, brimstone with muriate of soda, and the sulphate of soda and magnesia in a considerable quantity of warm water, but his favorite remedy is the *peristaltic persuaders* :—℞ Rhei ʒj ; Ipecac. gr. x; Ol. carui. gr. x; Acacia q. s. Ft. pil. xx ; cap. ij o. n.

*Hepatitis.* Dr. Chapman agrees with Annesley in deprecating the indiscriminate use of mercury in this disease, and has constantly observed the failure of that remedy to "operate specifically in any disease during the suppurative process." He adduces various evidence of the efficacy of taraxacum, and after mentioning the frequent dependence of hepatic affections on conditions of the duodenum, suggests the probability that the abuse of mercury may induce hepatitis, an opinion supported by the statement of Dr. Somervail, (a most respectable physician of the south of Virginia, who has practised medicine for nearly half a century, in that section of the country,) that, till the introduction of mercury, a comparatively modern event there, into the treatment of autumnal diseases, "hepatitis was hardly known, and subsequently, it has most widely prevailed." (p. 327.)

Dr. Chapman considers that he was the first to suggest the frequent dependence of jaundice on irritation of the duodenum.

*Diseases of the spleen.* Dr. Chapman offers some interesting remarks on the subject of "engorgement of the spleen," a state characterized by painful distension in the left hypochondrium, dull or lancinating pains, precordial uneasiness, difficult respiration, wildness and distraction, and a full active or slow and irregular pulse.

"Twice I have witnessed it from an immersion in a common cold bath, and in one instance, by that of the yellow springs of this vicinity, the water of which is of an exceedingly low temperature. Not uncommonly does it proceed from the exhaustion of long exposures to our summer heat, and particularly among our labouring people. Extraordinary exertion of any kind, by running, leaping, &c. may induce it, and I once knew it to follow the fatigue of standing in one position for a length of time. But usually it is brought on more slowly, by the gradual operation of miasmata, and though sometimes under such circumstances it attains to a considerable height, it is mostly of less severity." (p. 378.)

The remedy is depletion, followed by the administration of quinine.

We suspect that the author has not paid any attention to the peculiar condition of the circulating fluid induced by Bright's disease of the kidney, and that in consequence he refers changes thus induced to the spleen and other organs. This fact may also probably serve to explain such passages as the following:

"The aspect is strikingly anemic, or of a deadly white, the tendency to hemorrhage, or serous effusions, or both, very great, and sometimes to a fearful extent. The hemorrhagic disposition is indeed so considerable that copious flows of blood take place from the most trivial injuries, as a slight cut, or scratch of a pin, or the bite of a leech, &c." (p. 380-1.)

"More than once I have seen jaundice an attendant on gastro-enteric epilepsy, particularly with the lurid hue, and often in chlorotic affections, the complexion exhibiting all the gradations of the usual colours, as well as a waxy whiteness called icterus albus." (p. 359.)

We have certainly known epilepsy to arise from sympathy with gastric or intestinal irritation; and, under such circumstances, have found it subside after the use of prussic acid, and other remedies adapted to this condition; but we strongly suspect that the state referred to in the passage we have quoted is one dependent on renal disease. We have witnessed several cases in which epilepsy was evidently thus induced, and we have even known it to prove fatal without leaving traces of disease in any organ but the kidneys.

Judging from the indications afforded by this volume, with the exception of too devoted an allegiance to the sanguinary school of Rush, we should regard Dr. Chapman as a safe practitioner. Indeed, the simple fact that so long an experience has not destroyed his faith in the efficacy of appropriate treatment, furnishes a presumptive evidence of his practical skill.

Although our notice of the work has extended to some length, we have not exhausted the materials of interest which it contains. We wish those materials were arranged to better advantage; but the particles of opinion, numerous as they are, have not crystallized into form. The doctrines require to be organized by some pervading principle, and the whole expression calls for reconstruction. The author, we suspect, possesses much practical sagacity and diligence, but he has been too sparing of the pains requisite to make the work serviceable to the student. We leave it to be imagined what must be the composition of a volume in which the sentences which we have quoted are among the least obscure. It is painful to observe in the lectures of a respectable and intelligent physician so deplorable a deficiency of classical expression.

The attempt to conceal the defect by the introduction of passages from distinguished writers serves but to aggravate by contrast. Could Horace see the society into which his charms are introduced he would keenly feel the aptness of one of his own similes as he recognized the dolphins sporting in the woods. It is indeed difficult to imagine how a writer could be apparently familiar with so elegant a poet as Horace and yet so utterly avoid all imitation of that poet's terseness.

We are not fastidious critics. We acknowledge the difficulty of uniting a literary taste with the ample stores of knowledge which modern science expects us to obtain, but it is in the power of every writer to make himself intelligible, and we wish we could impress Dr. Chapman with an adequate sense of the duty. If he will consent to make the effort, his work may be so changed as to become really useful to the young practitioner. At present it scarcely contains a perspicuous sentence, and at every page requires much preliminary knowledge to enable the reader to guess the meaning.

Let us advise the author not to "bolt" but to digest his observations, and not to commence a sentence without some idea of the thoughts which it is intended to convey.

Notwithstanding the strictures which we have found it necessary to offer, we take leave of our American brother with real respect. He evinces for the most part great anxiety for truth, and an ardent love of humanity; and it is obviously his aim, beyond the ordinary standard, to distinguish those shades of difference in diseased conditions on the study of which we must mainly depend in regulating the niceties of practice and promoting the improvement of our art.

---

